# Enhanced removal of organic dyes from aqueous solutions by new magnetic HKUST-1: facile strategy for synthesis

**DOI:** 10.1038/s41598-023-45075-6

**Published:** 2023-10-20

**Authors:** Masoumeh Mohammadnejad, Niosha Mokhtari Nekoo, Sedighe Alizadeh, Soosan Sadeghi, Shokoofeh Geranmayeh

**Affiliations:** 1 Department of Analytical Chemistry, Faculty of Chemistry, Alzahra University, Tehran, Iran; 2 Department of Physical Chemistry and Nanochemistry, Faculty of Chemistry, Alzahra University, Tehran, Iran

**Keywords:** Magnetic properties and materials, Organic-inorganic nanostructures, Environmental sciences, Pollution remediation

## Abstract

A novel, magnetic HKUST-1 MOF based on MgFe_2_O_4_-NH_2_ was designed and synthesized in two steps and applied effective removal of malachite green (MG), crystal violet (CV), and methylene blue (MB) from water samples. Characterization of the newly synthesized MgFe_2_O_4_-NH_2_-HKUST-1 was performed by various techniques such as Fourier transform infrared spectroscopy, X-ray diffraction, Field emission scanning electron microscopy, Brunauer–Emmett–Teller, Thermal gravimetric analysis, and Vibration sampling magnetometry. Malachite green, crystal violet and methylene blue are toxic and mutagenic dyes that can be released into the water in different ways and cause many problems for human health and the environment. The removal of malachite green, crystal violet, and methylene blue from aqueous solutions was investigated using the magnetic HKUST-1 in this research. The effect of various parameters such as pH, amount of sorbent, dye concentration, temperature, and contact time on dye removal has been studied. The results showed that more than 75% of dyes were removed within 5 min. Adsorption isotherms, Kinetic, and thermodynamic studies were investigated. The results of this study show that adsorption capacity of the magnetic adsorbent is equal to 108.69 mg g^−1^ for MG, 70.42 mg g^−1^ for CV, and 156.25 mg g^−1^ for MB. This study shows the good strategy for the synthesis of the functionalized magnetic form of HKUST-1 and its capability for increasing the efficiency of the removal process of malachite green, crystal violet, and methylene blue from an aqueous solution.

## Introduction

Water is the source of life on Earth. The life and health of humans and other living depend on this valuable substance. Environmental problems and the release of organic dyes into water sources are important concerns in the world. Water pollution is one of the most serious and important problems in the world, which cause many problems in human society. Therefore, effective and efficient removal of pollutants from water is very important^1^. One of the main factors of environmental pollution is the growth of chemical industries. Many chemical industries such as paper, plastic, cosmetics, leather, printing, pharmaceutical, and textile use synthetic dyes to color their products. These dyes release into the water and cause the production of wastewater with dyes and organic contents. Malachite green, Crystal violet, and methylene blue are cationic triphenylmethane dyes that are used for different purposes in the industry^2,3^. Malachite green is an organic compound that is widely used in aquaculture to treat fungal attacks and protozoal infections and is used in other industries such as food, health, and textile industries^4,5^. However, the use of this dye has raised many concerns due to its reported toxic effects. Although the use of this dye is prohibited in several countries and is not approved by the United States Food and Drug Administration, it is still used in many parts of the world due to its low cost, availability, and efficiency^5,6^. Crystal violet is widely used in veterinary medicine as a biological stain, it is also used as a textile dye in textile processing^3^. Also, methylene blue is the most common material used to dye cotton, wool, and silk. It can cause eye burns that may cause permanent eye damage. In case of inhalation, it can lead to acute respiratory problems, while oral consumption causes a burning sensation and may cause nausea, vomiting, profuse sweating, and mental confusion^7,8^. These dyes are toxic, carcinogenic, mutagenic, and often stable against light and heat^9^. As access to clean water is essential for various activities, removing these dyes from water and wastewater requires an efficient sorbent. In the present study, the efficient removal of malachite green, crystal violet, and methylene blue was done using an efficient synthetic sorbent based on magnetic MOF. The dyes studied in this research are presented in Table 1.

**Table 1:** Chemical characteristics of organic dyes studied in this research.

Dye	IUPAC name	Molecular formula	Molecular mass (g/mol)	Chemical structure
Malachite green oxalate	[4-[[4-(dimethylamino)phenyl]-phenylmethylidene]cyclohexa-2,5-dien-1-ylidene]-dimethylazanium;2-hydroxy-2-oxoacetate;oxalic acid	C_52_H_54_N_4_O_12_	927.0	
Crystal violet	[4[bis[4(dimethylamino)phenyl]methylidene]cyclohexa-2,5-dien-1-ylidene]-dimethylazanium;chloride	C_25_H_30_ClN_3_	407.979	
Methylene blue	[7-(dimethylamino)phenothiazin-3-ylidene]-dimethylazanium;chloride	C_16_H_18_ClN_3_S	319.85	

Metal-organic frameworks (MOFs) are porous structures that are created from the coordinated bond between metal ions and organic linkers or bridging ligands^10^. The specific structural characteristics of MOFs include a wide range of particle sizes, high surface-to-volume ratio, high absorption tendency, controllability of particle size, and high absorption capacity^11^. Due to their high surface area, tunable structural properties, and thermal stability, MOFs are suitable for a wide range of applications, including catalysis^12^, gas storage^13^, enzyme carriers^14^, sensors^15^, and as a sorbent in the adsorption process has been studied^4^. The absorption capacity of MOFs to adsorb dyes is significant. One of the significant advantages of MOF compared to other sorbents is its structural diversity, the possibility of determining the size of the pores, and their properties by choosing different metal Ions and organic ligands in the synthesis stages^16^. HKUST-1 MOF contains Cu^2+^ units coordinated by four carboxylate groups and creates a highly porous cubic structure with a 3D network^17,18^. The important point in manufacturing HKUST-1 is the easy and reproducible synthesis of this MOF. MOFs can be used to treat dyes wastewater. MOFs are suitable materials for dye adsorption from wastewater with favorable performance compared to conventional sorbents^19^. Recently, for the better performance of MOFs, their composites are used, and in this study, a magnetic composite has been synthesized. Among the sorbents that have been synthesized so far for the removal of pollutants, magnetic sorbents have received much attention for absorbing dyes^20^.

Magnetic nanoparticles have attracted the attention of many researchers due to their extraordinary properties. They can be bound to MOFs by functionalization for the formation of a composite that has magnetic properties. Composites are a combination of two or more separate materials that have different properties than individual parts^21^.

HKUST-1 has attracted the attention of researchers in recent years due to its easy and cost-effective synthesis. On the other hand, it is difficult to separate MOFs from the mixed solution and it causes secondary pollution in the environment, and a centrifugal step is needed to separate them from the aqueous solution, so the use of MOF magnetic adsorbent allows easy separation with an external magnetic field and product recovery It will be easy and the operating cost will be more affordable. Also, the stabilization of adsorbent in aqueous environments using composite formation, a short time in the removal process, and acceptable adsorbent capacity are important in this study. Therefore, the magnetic HKUST-1 has been considered an accepted and efficient adsorbent for its easy separation.

In this study, the synthesized MgFe_2_O_4_ nanoparticles were functionalized by the –NH_2_ functional group to bind to the carboxylic acid group of HKUST-1 by hydrogen bonding. The new magnetic absorbent with the structure of MgFe_2_O_4_-NH_2_-HKUST-1 was studied to remove dye pollutants methylene blue, crystal violet, and malachite green. This compound shows adequate adsorption capacity, speed, and removal percentage for removing dyes from wastewater. The effective factors, isotherms, kinetics, and thermodynamics of the adsorption removal process were investigated.

## Experimental

### Materials and instruments

Malachite green (MG), Crystal violet (CV), Methylene blue (MB), Iron (III) nitrate nonahydrate Fe(NO_3_)_3_·9H_2_O, Magnesium nitrate hexahydrate Mg(NO_3_)_2_·6H_2_O, Copper(II) nitrate trihydrate Cu(NO_3_)_2_·3H_2_O, benzene-1, 3,5-tricarboxylate (BTC), Sodium acetate C_2_H_3_NaO_2_, Ethylene glycol C_2_H_6_O_2_, Ethanolamine C_2_H_7_NO, Ethanol C_2_H_5_OH, Hydrochloric acid HCl were purchased from Merck and Sigma Aldrich company.

X-ray powder diffraction (XRD) measurements were performed using a Philips X’pert diffractometer with monochromatic Cu-Kα radiation. A glass pH electrode (Metrohm 713 pH-meter) was used for pH measurements. The morphology of samples was studied by field emission scanning electron microscopy (FE-SEM) KYKY-EM 3200 with gold coating. Fourier transform infrared spectroscopy (FT-IR) spectra were obtained on Equinox 55 BRUKER model FT-IR spectrometer. The absorbance measurement was carried out by double-beam UV–Vis spectrophotometer using a 1 cm quartz cell (Perkin-Elmer, Lambda 35, USA). BET (Belsorp mini II, Microtrace bel Corop), TGA (Bahr, Germany), zeta potential (SZ-100z), and vibrating sample magnetometer (Lake Shore Cryotronics-7407) analysis were done.

### Synthesis of MgFe_2_O_4_-NH_2_

**Scheme 1:**
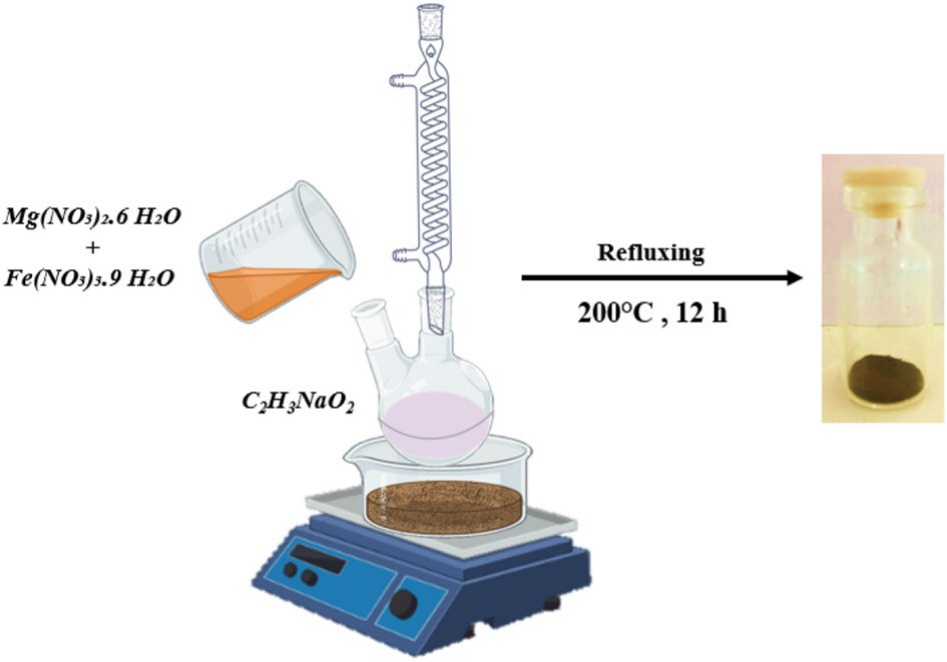
The magnetic nanoparticle synthesis process.

10 mmol of CH_3_COONa as dissolved in 6.5 mL Ethylene glycol and heated to 100 °C under a magnetic stirring and refluxing system in the sand bath for 15 min. A solution of 0.66 mmol of Mg(NO_3_)_2_·6H_2_O and 1.3 mmol of Fe(NO_3_)_3_·9H_2_O dissolved in 3.5 mL ethylene glycol was poured into the preheated solution rapidly. The mixture was stirred for 30 min, and then 2.3 mL of ethanolamine was added. The mixture was heated to 200 °C and maintained for 12 h and then, the system was cooled to room temperature naturally. The black precipitate was collected by a magnetic separator, washed with double distilled water excessively, and then ethanol. The final product, the MgFe_2_O_4_-NH_2_ nanoparticle, was dried at 70 °C for 4 h^22^ (Scheme 1).

### Synthesis of MgFe_2_O_4_-NH_2_-HKUST-1 composite

**Scheme 2:**
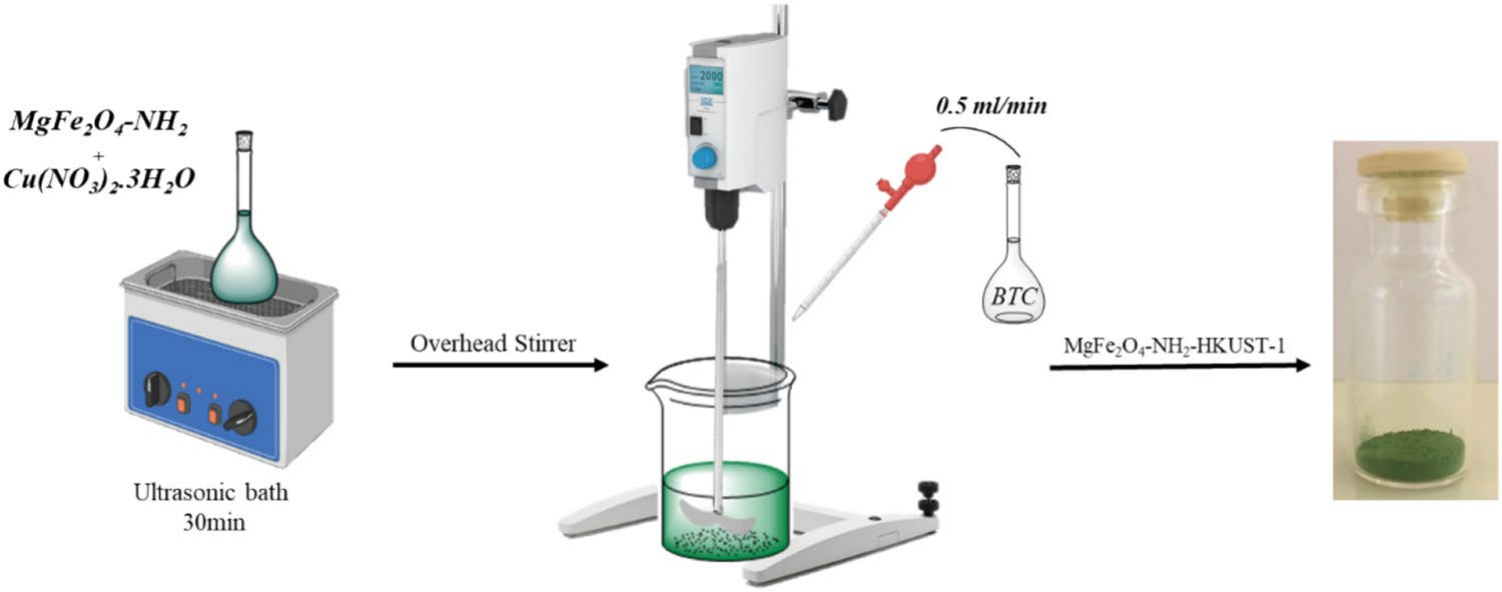
The magnetic composite synthesis process.

0.05 g of the synthesized nanoparticle (MgFe_2_O_4_-NH_2_) and 2.4 mmol of copper nitrate were dissolved in 25 ml of ethanol and placed in an ultrasonic bath for 30 min. Then 1 mmol of BTC was dissolved in 25 ml of ethanol and added to the previous solution at a rate of 0.5 mL/min under mechanical stirring for 1 h. Then, the final product (green precipitate) was washed several times with ethanol separated by an external magnet, and then dried under a temperature of 50 °C for 4 h in a vacuum oven (Scheme 2).

### Adsorption process

**Scheme 3:**
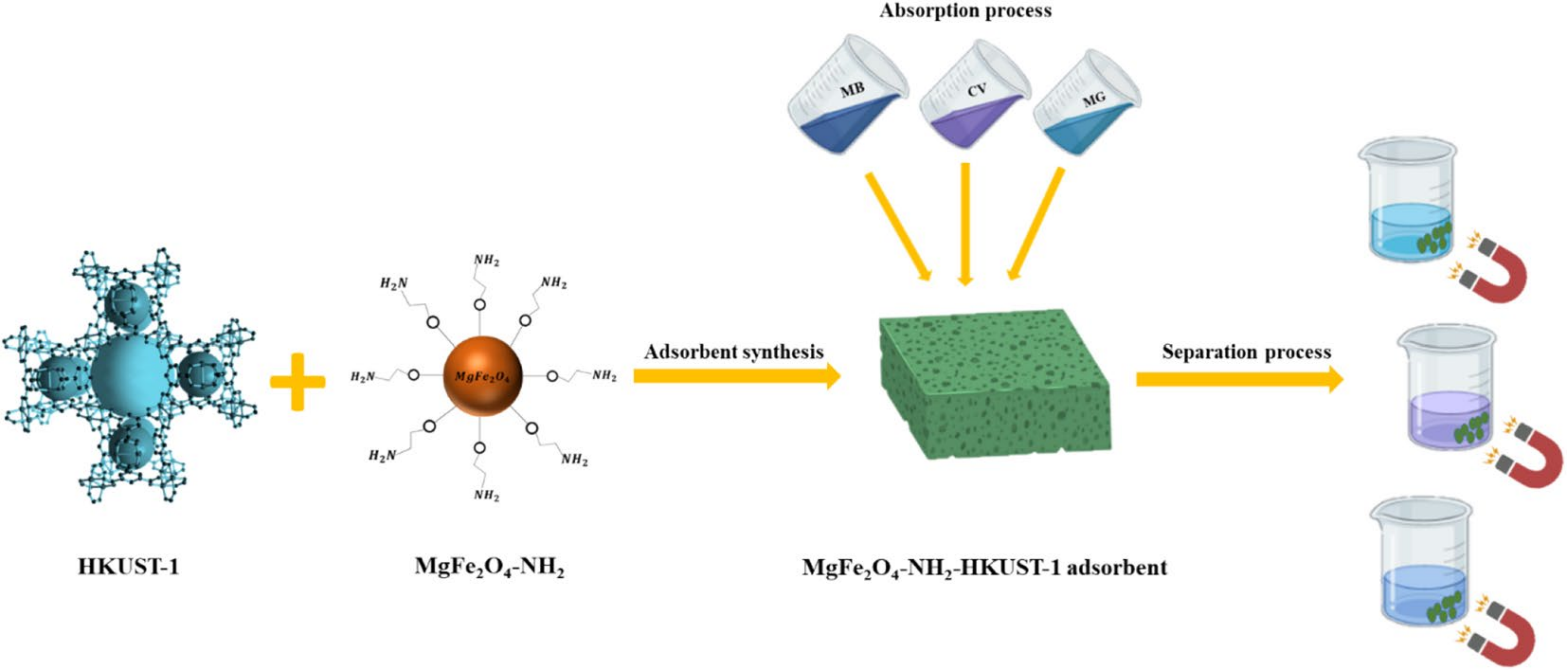
The process of removing organic dyes and separating the sorbent from the solution.

Different solutions of dyes were prepared by dissolving appropriate amounts of MG, CV, and MB in the range of 0.18–9.27 mg L^−1^, 0.81–6.94 mg L^−1^, and 0.63–5.43 mg L^−1^, respectively, in distilled water. The calibration curves were obtained by measuring the absorbance for MG at 620 nm, CV at 590 nm, and MB at 664 nm. The adsorption process was carried out by the addition of 10 mg, 5.5 mg, and, 1 mg of sorbent separately to the 5 mL solutions with different concentrations of MG, CV, and, MB (Scheme 3).

The solutions including the sorbent were mixed with a magnetic stirrer for 5 min for all dyes. Subsequently, the sorbent was separated by an external magnet, and the remaining MG, CV, and MB concentrations were calculated by the following equation:

1 $$ Removal \;(\% ) = \left( {1 - \frac{A}{{A_{0} }}} \right) \times 100 $$

where A is the adsorption of the analyte after adding the sorbent and A_0_ is the initial adsorption of the solution.

## Result and discussion

### Characterization of MgFe_2_O_4_-NH_2_-HKUST-1 composite

For characterization of prepared magnetic composite, FT-IR spectra (4000–400 cm^−1^) in KBr pellet, X-ray powder diffraction (XRD) measurements, field emission scanning electron microscopy (FE-SEM), Brunauer–Emmett–Teller (BET), Thermal gravimetric analysis (TGA) and Vibrating sample magnetometer (VSM) were performed.

#### FT-IR analysis

**Figure 1:**
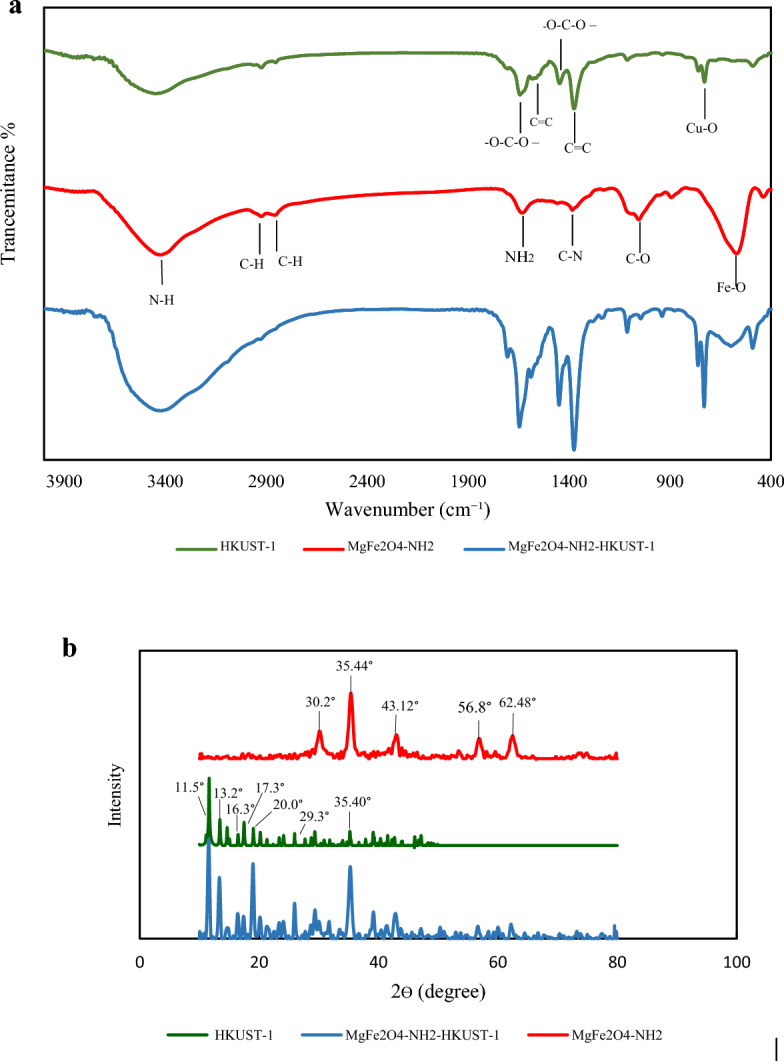
(**a**) FT-IR spectra of HKUST-1, MgFe_2_O_4_-NH_2_ and MgFe_2_O_4_-NH_2_-HKUST-1, (**b**) XRD pattern of MgFe_2_O_4_-NH_2_, H KUST-1, and MgFe_2_O_4_-NH_2_-HKUST-1.

The Fourier transform infrared (FT-IR) spectra of HKUST-1, MgFe_2_O_4_-NH_2_, and MgFe_2_O_4_-NH_2_-HKUST-1 have been shown in Fig. 1a.

In the FT-IR spectra related to HKUST-1 Bands in 1447 cm^−1^ and 1639 cm^−1^ are attributed to –O–C–O– groups, and the bands in 1375 cm^−1^ and 1565 cm^−1^ are attributed to the C=C stretching vibration of the BTC ligand. The band in 680 cm^−1^ is related to the Cu–O bond^23^. The broad peak at 3420 cm^−1^ can be attributed to the -OH of water molecules^24^. In the FT-IR spectra related to MgFe_2_O_4_-NH_2_ the bands in 2923 cm^−1^ and 2856 cm^−1^ are attributed to the stretching vibration of the C–H bond in ethanolamine or ethylene glycol. The band in 1054 cm^−1^ can be attributed to the overlap of the C–O bond with the C–N stretching vibration, which is a sign of the binding of amine groups on the MgFe_2_O_4_-NH_2_ nanoparticle^22^, The band in 570 cm^−1^ is related to the Fe–O bond. Also, the bands in 1383 cm^−1^, 1630 cm^−1^, and 3420 cm^−1^ are related to C–N stretching vibration, NH_2_ scissor bending vibration, and N–H stretching vibration, which indicates the presence of ethanolamine molecules on the nanoparticle surface^25^.

Therefore, the peaks appearing in the spectrum of HKUST-1 and MgFe_2_O_4_-NH_2_ can be seen in the spectrum corresponding to the synthesized composite.

#### XRD analysis

To investigate the phase purity of the prepared sorbent, powder X-ray diffraction (XRD) was carried out (Fig. 1b). In the XRD pattern related to MgFe_2_O_4_-NH_2_, peaks at the angles of 2θ = 30.2°, 35.44°, 43.12°, 56.8°, and 62.48° are the characteristic peaks of the synthesized compound^25^. In the XRD pattern related to HKUST-1, the peaks in the angles of 2θ = 11.5°, 13.2°, 16.3°, 17.3°, 20.0°, 29.3°, 35.40° are the characteristic peaks of this structure^23^. The peaks appearing in pattern of HKUST-1 and MgFe_2_O_4_-NH_2_ can be seen in the pattern of the synthesized composite.

#### FE-SEM micrographs

**Figure 2:**
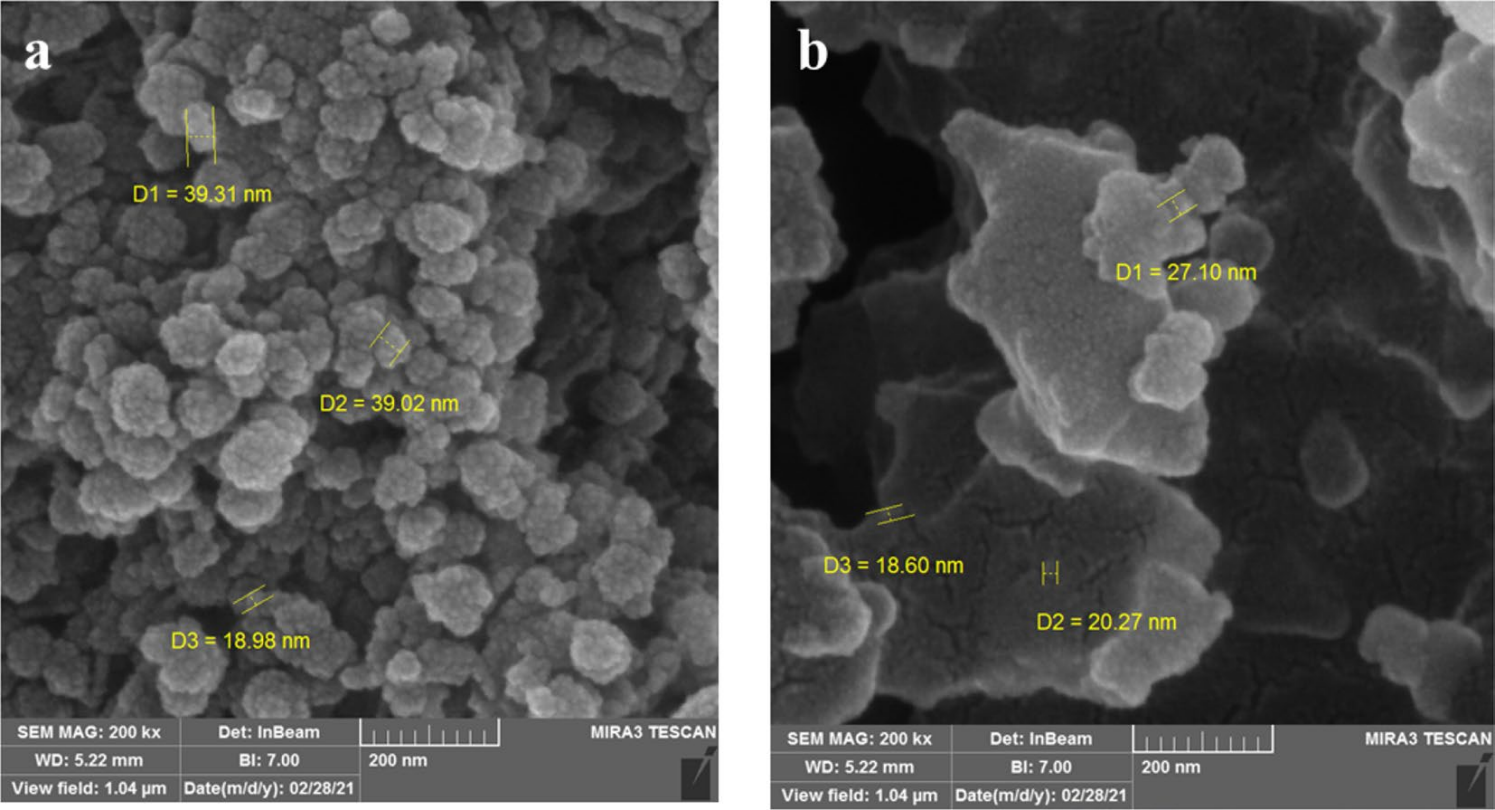
(**a**) FE-SEM of MgFe_2_O_4_-NH_2_, (**b**) FE-SEM of MgFe_2_O4-NH_2_-HKUST-1 magnetic composite.

FE-SEM was also carried out to observe the morphology of the MgFe_2_O_4_-NH_2_-HKUST-1. In Fig. 2a, FE-SEM images of metal oxide nanoparticles MgFe_2_O_4_-NH_2_ are given. These images show that the synthesized nanoparticles are spherical with an approximate diameter of 40 nm. In Fig. 2b, nanocomposite MgFe_2_O_4_-NH_2_-HKUST-1 is given which can represent the synthesized magnetic composite framework.

#### BET analysis

**Figure 3:**
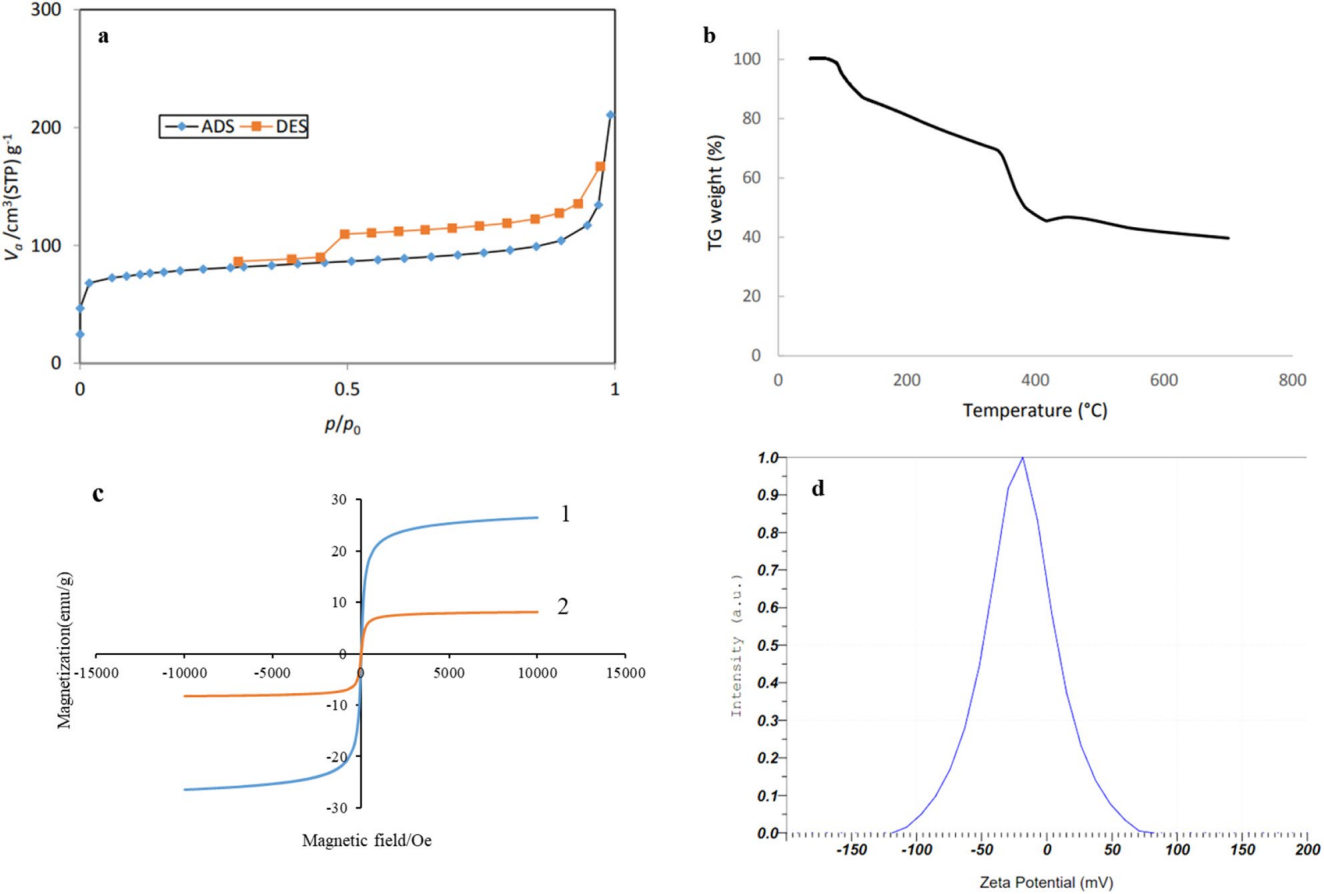
(**a**) Nitrogen gas adsorption–desorption curve for the synthesized composite. (**b**) The thermal analysis of MgFe_2_O_4_-NH_2_-HKUST-1. (**c**) Magnetic analysis spectrum of (1) MgFe_2_O_4_-NH_2_ magnetic nanoparticle, (2) MgFe_2_O_4_-NH_2_-HKUST-1 magnetic composite. (**d**) Zeta potential of magnetic composite MgFe_2_O_4_-NH_2_-HKUST-1.

The surface properties of the MgFe_2_O_4_-NH_2_-HKUST-1, such as the surface area, and pore diameter were also examined using BET surface area analysis. The specific surface area of the MgFe_2_O_4_-NH_2_-HKUST-1 composite evaluated was obtained at 297.13 m^2^ g^−1^. Also, average pore diameter distribution was investigated using the Barret–Joyner–Halenda (BJH) method. The average pore diameter of the synthesized composite was reported to be 4.26 nm (Table 2). The adsorption–desorption curve of nitrogen gas for the synthesized composite shows I/IV mixed type isotherm, which means MgFe_2_O_4_-NH_2_ nanoparticles are with microporous and mesoporous structures at the same time, as shown in Fig. 3a.

**Table 2:** Information about the porosity of the synthesized composition.

Compound	Average pore diameter (nm)	Specific surface area (m^2^ g^−1^)
MgFe_2_O_4_-NH_2_-HKUST-1	4.26	297.13

#### TGA analysis

The thermal stability of the MgFe_2_O_4_-NH_2_-HKUST-1 composite was estimated via TGA. The TGA profile depicted three weight loss steps in the tested temperature range of 50–700 °C (Fig. 3b). The first weight loss %20 appeared in the temperature range of 80–150 °C, which probably indicates the removal of surface water or residual solvent and physisorbed and chemisorbed H_2_O molecules in the sample. In the second stage, in the temperature range of 150–420 °C, organic binders begin to degrade and eventually lead to the complete collapse of the composite, and the remaining mass in this stage reaches 45% of the initial mass. In the third stage, with an increase in temperature from 420 °C, an increase in mass of about 2% by weight is observed. This increase in mass can be due to the formation of some oxides that are not stable at higher temperatures and gradually decompose. Also, the approximate stability of the sample mass in the range of 40% of the initial mass can be attributed to the presence of CuO and Fe_3_O_2_ compounds^23,26–28^.

#### VSM analysis

To study the magnetic behavior of MgFe_2_O_4_-NH_2_ nanoparticles and MgFe_2_O_4_-NH_2_-HKUST-1 composite, magnetization measurements were performed by VSM. As seen in (Fig. 3c (1), the value of saturation magnetic (Ms) was 28.48 emu g^−1^ for MgFe_2_O_4_-NH_2_ nanoparticles and according to Fig. 3c (2), the maximum saturation magnetization of the MgFe_2_O_4_-NH_2_-HKUST-1 composite was obtained 8.13 emu g^−1^, which is enough to quickly collect it by a strong magnet. from a large volume of water. The amount of saturation magnetization of magnetic composite has decreased compared to magnetic nanoparticles, which can be caused by an increase in the thickness of the non-magnetic component. Also, due to the reduction of the coercivity force (H_C_), it is concluded that the synthesized sorbent has superparamagnetic properties.

#### Zeta potential

To check the surface charge of the synthesized composite, the zeta potential was used. The zeta potential of the sorbent is one of the factors that can affect the adsorption capacity. According to Fig. 3d, the results showed that the surface charge of the synthesized sorbent is negative and equal to − 20.5 mV, so MG, CV, and MB cationic dyes are adsorbed on the surface of the sorbent**.**

### Optimization of experimental conditions for dyes removal

To obtain more effective adsorption of MG, CV, and MB, the effect of adsorption conditions was investigated and optimized. These parameters are initial solution pH, amount of sorbent, and contact time.

#### Initial solution pH

**Figure 4:**
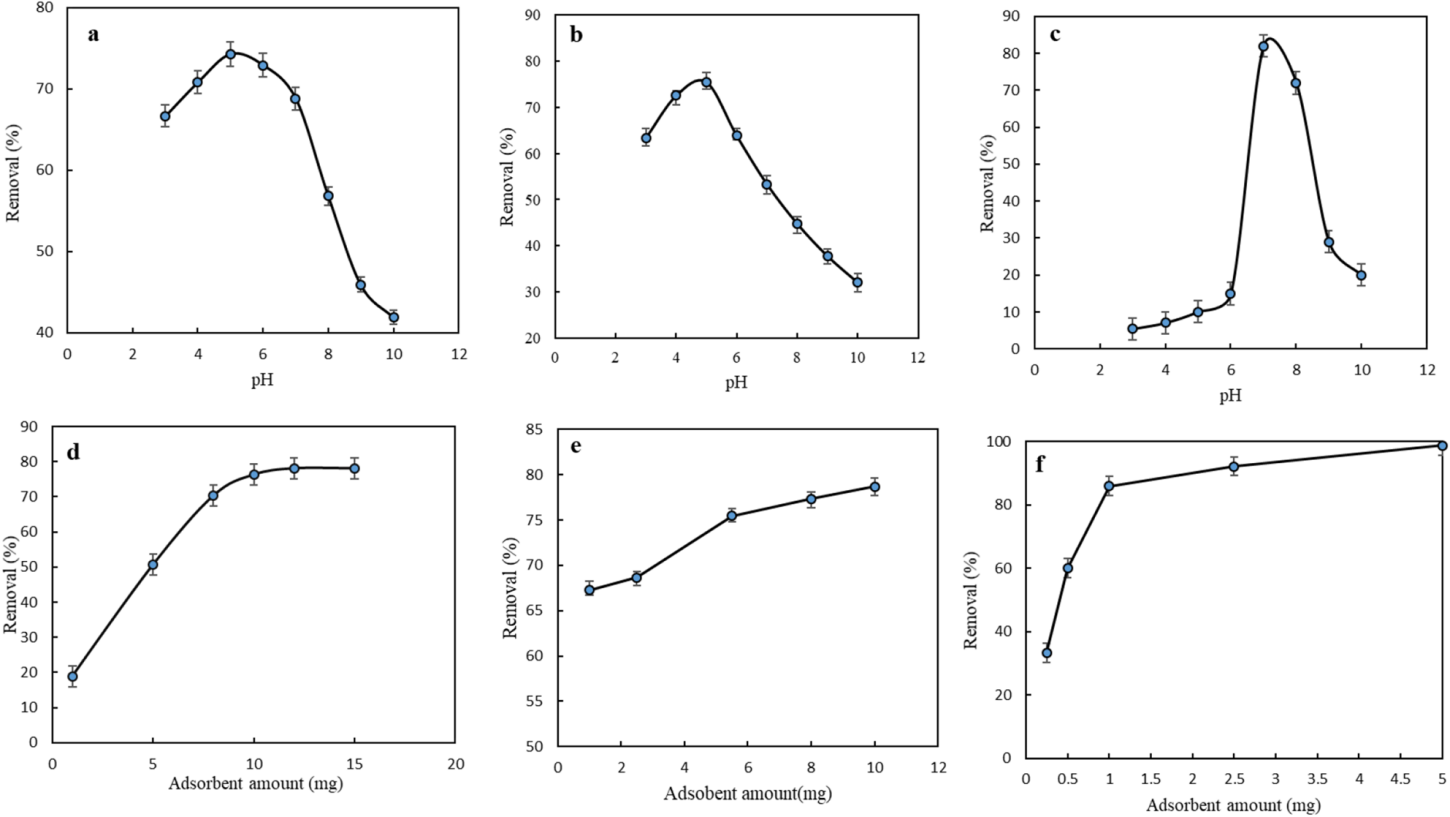
The effect of pH on the removal process of (**a**) MG, (**b**) CV, and (**c**) MB by the sorbent in the range of 3.0–10.0 and the graph of the removal percentage of (**d**) MG, (**e**) CV, and (**f**) MB from the solution according to the weight of the sorbent.

In this procedure, adsorption experiments of MG, CV, and, MB were done within the solution pH ranging from 3.0 to 10.0, and %removal was calculated (Fig. 4a–c). The adsorption capacities of MgFe_2_O_4_-NH_2_-HKUST-1 for MG, CV, and MB increased with the solution pH increasing to 5.5, 5, and 7. However, the adsorption capacity decreased up to 10. According to the p*K*_a_ and cationic nature of these dyes at the mentioned pH and also based on the zeta potential analysis that shows the sorbent surface is negative it can be concluded these cationic dyes are protonated at the tested pH and are probably electrostatically adsorbed by the sorbent.

#### Amount of sorbent

The amount of sorbent could directly affect the adsorption capacity. To obtain the optimal adsorption conditions, experiments were carried out by the addition of 1–12.0 mg, 1–10 mg, and 0.25–5 mg of sorbents to a series of 5 mL of 1.3 × 10^−5^ mol/L solution of MG and CV and MB, respectively. The optimum weight of sorbent for the removal of MG, CV, and MB has obtained 10 mg, 5.5 mg, and 1 mg, respectively as shown in Fig. 4d–f.

#### Adsorption time

The adsorption rate is an important factor. Absorbance spectra for MG, CV, and MB were recorded by time in the presence of sorbent. The optimal time for three dyes was 5 min, with a high removal percentage which is one of the excellent features of the synthesized sorbent.

#### Stability and reproducibility

According to the tests, the synthesized adsorbent can be used for at least 5 cycles without significant decrease in its efficiency. Also to check the reproducibility, adsorbents were synthesized in different times and removal experiments performed under the mentioned optimal conditions. The reproducibility of the adsorbent for 3 dyes is reported in Table 3.

**Table 3:** Reproducibility tests.

Test no	Analyte	pH	Amount of sorbent (mg)	Adsorption time (min)	% Removal	% RSD
1	MG	5.5	10	5	75.6	0.88
2					74.7	
3					75.8	
4					76.3	
1	CV	5	5.5	5	76.6	0.75
2					75.8	
3					75.3	
4					75.5	
1	MB	7	1	5	82.8	1.04
2					84.9	
3					83.5	
4					83.6	

### Adsorption capacity and isotherm

**Figure 5:**
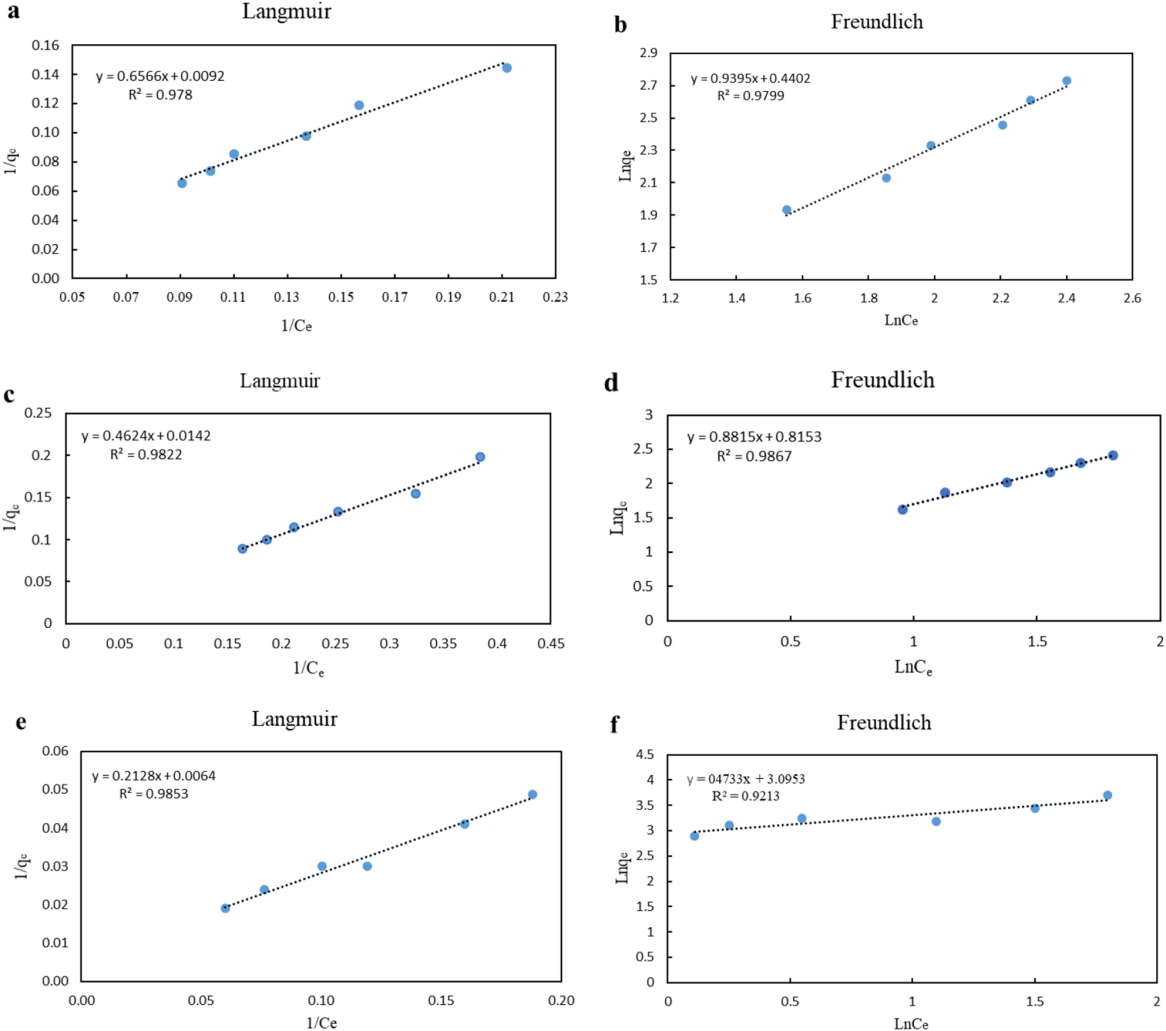
Langmuir and Freundlich isotherm diagram of MG (**a**,**b**), CV (**c**,**d**), and MB (**e**,**f**).

To describe the adsorption mechanism for MG, CV, and MB, the values of 1/q_e_ versus 1/C_e_ were plotted for the Langmuir isotherm. The values of Ln q_e_ versus Ln C_e_ were plotted for the Freundlich isotherm. Equations (2) and (3) express the linear form of Langmuir and Freundlich isotherms, respectively:

2 $$ \frac{1}{{q_{e} }} = \frac{1}{{q_{max} K_{L} C_{e} }} + \frac{1}{{q_{max} }} $$

3 $$ Lnq_{e} = LnK_{F} + \frac{1}{n}LnC_{e} $$

where C_e_ (mg L^−1^) represents the equilibrium concentration of MG, CV, and MB; q_e_ (mg g^−1^) is the equilibrium adsorption capacity; q_max_ (mg g^−1^) is the maximum adsorption capacity; K_L_ (L mg^−1^ or L mol^−1^) is Langmuir constant represents the adsorption energy and K_F_ and n are the Freundlich constants^29^. Figure 5 and Table 4 show Langmuir and Freundlich isotherm diagrams and results for MG, CV, and MB.

**Table 4:** Langmuir and Freundlich isotherm constants of MG, CV, and MB constants.

Constants	MG	CV	MB
Langmuir equation			
R^2^	0.9780	0.9822	0.9853
q_max_ (mg g^−1^)	108.69	70.42	156.25
K_L_ (L mg^−1^)	0.01	0.03	0.03
R_L_	0.91	0.85	0.97
Freundlich equation			
R^2^	0.9799	0.9867	0.9213
K_F_ (mg g^−1^) (L mg^−1^)^1/n^	1.55	2.25	22.08
n	1.06	1.13	2.12

### Adsorption kinetics

Adsorption rate is an important characteristic for the evaluation of sorbents. Fast adsorption onto MgFe_2_O_4_-NH_2_-HKUST-1 occurred in the initial adsorption stage. For example, more than 75% of dyes were removed within 5 min when the initial MG concentration was 10^−5^ mol/L with 10 mg of sorbent and CV concentration was 1.3 × 10^−5^ mol/L with 5.5 mg of sorbent, and MB concentration was 1.3 × 10^−5^ mol/L with 1 mg of sorbent. To further analyze and calculate the kinetic parameters, the experimental data were fitted by two kinetic models: the pseudo-first-order Eq. (4) and pseudo-second-order Eq. (5) kinetic models^30^:

4 $$ Log(q_{e} - q_{t} ) = Logq_{e} - \frac{{k_{1} t}}{2.303} $$

5 $$ \frac{t}{{q_{t} }} = \frac{1}{{k_{2} q_{e}^{2} }} + \frac{t}{{q_{e} }} $$

where q_t_ (mg g^−1^) and q_e_ (mg g^−1^) are the amounts of MG, CV, and MB adsorbed at time t (min) and the amount at adsorption equilibrium, respectively, and k_1_ (min^−1^) and k_2_ (g mg^−1^ min^−1^) are the pseudo-first-order and pseudo-second-order rate constants, respectively.

The fitting results (Table 5) show that the adsorption of MG, CV, and MB onto MgFe_2_O_4_-NH_2_-HKUST-1 could be well described by the pseudo-second-order kinetic model.

**Table 5:** Pseudo-first-order and pseudo-second-order kinetic model.

Kinetic model	MG	CV	MB
Pseudo-first-order			
k_1_ (min^−1^)	1.26	0.34	1.89
R^2^	0.917	0.987	0.921
Pseudo-second-order			
k_2_ (g mg^−1^ min^−1^)	4.94	3.68	0.765
R^2^	0.996	0.999	0.993

### Adsorption thermodynamics

The thermodynamic parameters, standard free energy change (ΔG°, kJ mol^−1^), enthalpy change (ΔH°, kJ mol^−1^), and entropy change (ΔS°, J mol^−1^ K^−1^) related to the adsorption were determined. These parameters can be calculated with the following equations (Eqs. 6, 7).

6 $$ \Delta G^\circ = - RTLn\left( {\frac{{q_{e} }}{{C_{e} }}} \right) $$

7 $$ \Delta G^\circ = \Delta H^\circ - T\Delta S^\circ $$

where, T (K) is the temperature in Kelvin, R (8.314 J mol^−1^ K) is the universal gas constant and K (L mol^−1^) is the thermodynamic equilibrium constant for the adsorption process calculated from the ratio of the equilibrium adsorption capacity (q_e_) to the equilibrium concentration of the solution (C_e_).

The thermodynamic parameters were determined at 21,116 and 18,217 kJ mol^−1^ for ΔH and 75.493 and 67.856 J mol^−1^ K^−1^ for ΔS for MG and CV respectively. The results confirm an endothermic nature for MG and CV, as the temperature increases, the removal percentage of MG and CV increases. The adsorption process of these two dyes is a possible and spontaneous process. The thermodynamic parameters were determined − 7102.3 kJ mol^−1^ for ΔH and 6.05 mol^−1^ K^−1^ for ΔS for MB. Unlike MG and CV, the adsorption of MB decreased with increasing temperature and the adsorption process was exothermic.

### The mechanism of the adsorption process

The organic dye adsorption process occurs because the adsorbent’s surface particles are not in the same environment as the particles within the bulk. Inside the MgFe_2_O_4_-NH_2_-HKUST-1, all of the forces acting between the particles are mutually balanced, but on the surface, the particles are not surrounded by atoms or molecules of their kind, and thus they have unbalanced or residual attractive forces. These adsorbent forces are responsible for attracting dyes to their surface. Some studies have shown that dye adsorption in MOFs is controlled by electrostatic interactions^31^. Therefore, the pH of the solution is expected to affect the amount of dye adsorbed as a result of the presence or absence of electrical charges on the dye molecules and the adsorbent surface. These dyes have a positive charge at the mentioned optimal pH and according to the results of the zeta potential analysis and the negativity of the surface charge of the absorbent, the adsorption process occurs due to the electrostatic interaction between the cationic dye and the absorbent surface.

## Sorbent efficiency in removing other dyes

**Figure 6:**
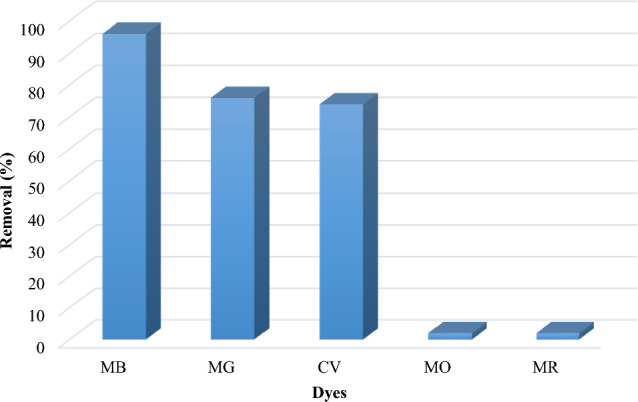
Comparison of the performance of the synthetic composite for removing different dyes.

Industrial effluents and aqueous solutions are a mixture of various pollutants, including organic dyes. The selectivity of a sorbent is important if it can separate a group of specific dyes from other pollutants, and it is one of the features that are considered in the manufacture of sorbents. In this study, magnetic composite MgFe_2_O_4_-NH_2_-HKUST-1 can absorb cationic dyes such as malachite green, crystal violet, and methylene blue while two anionic dyes, metal orange, and methyl red, are not adsorbed on the sorbent surface. This feature can be an advantage for the studied absorber. Figure 6 shows the high adsorption capacity of the sorbent used for cationic dyes, while the adsorption capacity for the other two anionic pollutants is very small.

## Comparison with other studies

The synthesized magnetic adsorbent was compared with some adsorbents to remove of MG, CV, and MB and shown in Table 6. As it is known, the synthesized adsorbent shows a good absorption capacity and extremely short time of 5 min for the absorption process. Also, the ability to easily separate the adsorbent with an external magnet is a special feature of the synthesized adsorbent compared to non-magnetic adsorbents.

**Table 6:** Comparison of adsorption capacities of MG, CV, and MB using various MOFs obtained in the present work and reported in the literature.

Dye	Adsorbent	Time (min)	q_max (mg/g)_	References
MG	MIL-53(Al)	30	81.2	^32^
	Fe-BTC	30	177	^33^
	MgFe_2_O_4_-NH_2_-HKUST-1	5	108.69	Present study
CV	SH NH_2_-MIL-125 (Ti)	180	129.87	^34^
	H_2_dtoaCu	100	165.83	^35^
	MgFe_2_O_4_-NH_2_-HKUST-1	5	70.42	Present study
MB	HKUST-1	40	101	^36^
	Amin-MOF-Fe	180	312.5	^37^
	MgFe_2_O_4_-NH_2_-HKUST-1	5	156	Present study

## Conclusion

In this research, magnetic adsorbent MgFe_2_O_4_-NH_2_-HKUST-1 was synthesized as an efficient adsorbent in absorbing malachite green, crystal violet, and methylene blue. The results showed that the synthesized adsorbent has a negative surface charge that creates an electrostatic attraction between the cationic dyes and the adsorbent surface. The results of this study showed that the adsorption capacity of the magnetic adsorbent is equal to 108.69 mg g^−1^ for MG, 70.42 mg g^−1^ for CV, and 156.25 mg g^−1^ for MB. It was observed that malachite green, crystal violet, and methylene blue respectively at pH = 5.5, 5, and 7 at the optimum time of 5 min, and amounts of adsorbent 10, 5.5, and 1 mg were removed more than 75%. The synthesized adsorbent has a high potential to remove dyes in a very short contact time. In addition, this absorbent with its magnetic property is very quickly and easily collected by an external magnet from the working environment, which easily eliminates the difficult and time-consuming step of separating the solid phase from the solution and using a centrifuge. So the process of absorbing by this absorbent is an efficient, fast, and economical process. MgFe_2_O_4_-NH_2_-HKUST-1 composite as a magnetic adsorbent can be a promising future for environment-based processes.

## Data Availability

All data generated or analyzed during this study are included in this published article.
